# Supplementary material for: Exploring dynamic solvation kinetics at electrocatalyst surfaces
Source: Nat Commun. 2024 Sep 18;15:8204. doi: 10.1038/s41467-024-52499-9 (PMC11411097; doi:10.1038/s41467-024-52499-9)
Supplement: Supplementary file 1 — Supplementary Information [file 41467_2024_52499_MOESM1_ESM.pdf]

The PDF file includes:

Supplementary Notes 1 - 4
Supplementary Figures 1 - 15
Supplementary References

Supplementary Note 1

Relaxation time constants

Even in absence of mass transport limitations and local pH changes, there exists a broad frequency spectrum of capacitances at liquid-solid interfaces[1]. Fast electronic polarization, electron and ion transfer and ordering of the first few water molecules at the electrode surface[2] (~ THz, ps) induce dipole relaxation time constants over the inner-Helmholtz layer[3,4] (GHz-MHz, ns), which can lead to ms-time constants for double layer charging in real electrochemical cells, depending on the dimension of the cell[5,6]. Additional factors, such as ion accumulation[7,8] and (pseudo-capacitive) changes in the coverage and oxidation state changes of metal (oxide) sites can lead to additional slow time constants (MHz – 50 Hz, ms-s) and, potentially, influence Faradaic processes (~ < 50 Hz). We stress that electronic polarization, electric fields and water ordering, as well as, ionic and electronic charge transfer are all much faster processes (~1ps) than those one can temporally resolve exclusively based on traditional electrochemical methods alone[2,9]. In Fig. 2, we observe the separation of double layer charging (ms-time scale) from electrosorption currents (analyzed *via* the steady-state Arrhenius equation).

Supplementary Note 2

Assessing the goodness of linear regression models for temperature dependent studies

Temperature-dependent analysis usually involves linearizing the Arrhenius equation and performing a least-squares linear regression of the $\log_{10}$ of the current density vs the inverse of the temperature in Kelvin, extracting from the slope E_A and from the origin intercept $\log_{10}A$. The goodness of the regression is usually assessed by the R-squared (R^2) coefficient, which ranges from 0 to 1, and which gives a relative measure of how good the model explains the variance of the dependent variable, i.e. how good the model predicts the variation of the dependent variable (y) given a change in the independent variable (x). However, it falls short in predicting the precision of the model's prediction. For this, the standard error of the regression (S) provides an absolute measure of the distance between data points and the regression line, the smaller S is the closer the fitted values are to the actual data points. The distinction between R^2 and S becomes especially relevant when considering fitting data with small variations in the dependent variable such as when the relative changes of current with temperature are small, e.g., when the activation energies are close to zero. For these cases, the R^2 coefficient will be close to zero since the variation in the independent variable (temperature) does not correspond to a variation in the dependent variable (current). Consider the two Arrhenius linear regressions shown in Supplementary Figure 15a. While the orange regression shows a high R^2 coefficient and high slope value, its absolute standard error is comparable to another regression with an R^2 coefficient close to zero with a small slope value. This is especially relevant for this work since many of the linear regressions are done for temperature dependent analysis with small positive, negative, or close to zero activation energies. For this reason, in addition to using R^2, the goodness of the fit should be done considering S, which is plotted as error bars in the $\log_{10}A$ E_A plots (Supplementary Figure 15b).

Supplementary Note 3

Impedance measurements

As in our previous work[10], we extract the real part of the frequency (ω) dependent capacitance according to $C_R(\omega) = -Z(\omega)_I \cdot (\omega \cdot |Z(\omega)|^2)^{-1}$, where $Z(\omega)_I$ is the imaginary impedance and $|Z(\omega)|$ the absolute impedance value (Supplementary Figure 5-6), that informs on the reversible

amount of energy stored at the interface, according to a simple RC series circuit[11,12]. Here, we refrain from fitting the data with traditional circuit elements, where the Faradaic current is only dependent on the charge transfer resistance (R) and not promoted by changes in the capacitance (C). Due to low series resistance (~1-2 Ω) (Supplementary Figure 7) and absence of diffusional Warburg limitations, we extract changes in the capacitance reliably between 50 kHz – 1Hz.

The challenge of studying fast OH/H electrosorption kinetics has previously been encountered in high frequency impedance (~10 MHz) studies[13,14], that concluded that these processes are too fast to be studied with electrochemical methods alone. However, other impedance studies were able to determine the charge transfer resistance for hydrogen underpotential deposition in alkaline media[15]. Furthermore, electrosorption kinetics were studied with fast cyclic voltammetry (1000 V/s over a ~ 1V window[16,17]. The resulting frequency range is consistent with the results in Fig. 2.

Supplementary Note 4

Delineation of mass transport from kinetics

The increase in the pre-exponential factors with increasing bias cannot be explained by mass transport limitations: (i) All currents were generally ≤ 1.5 mA cm^{-2} for an electrolyte concentration of 0.1 M KOH. (ii) The Arrhenius fits (neglecting mass transport) reach $R^2 > 0.999$ values. (iii) The impedance does not display any simple Warburg diffusion impedance over the whole studied potential and current range. (iv) The potential dependent changes of the low series resistance are negligible. (v) For all reactions, starting at very low current densities (<< 1 mA cm^{-2}), A and E_A increase and then saturate at higher currents and potentials. For the HER, at higher bias the kinetics start to approach Butler-Volmer kinetics (although differences remain even in that regime). Mass transport limitations, such as local pH changes or dissolved O_2 transport, and catalyst deactivation lead to increasing deviation with increasing bias. Bias dependent capacitive charging of metal interfaces and bias and time dependent coverage of acid-base sites are fundamentally distinct from local pH changes and (bulk) transport limitations. Conversely, we refrain from assigning any of these dynamic properties to a "surface pH".

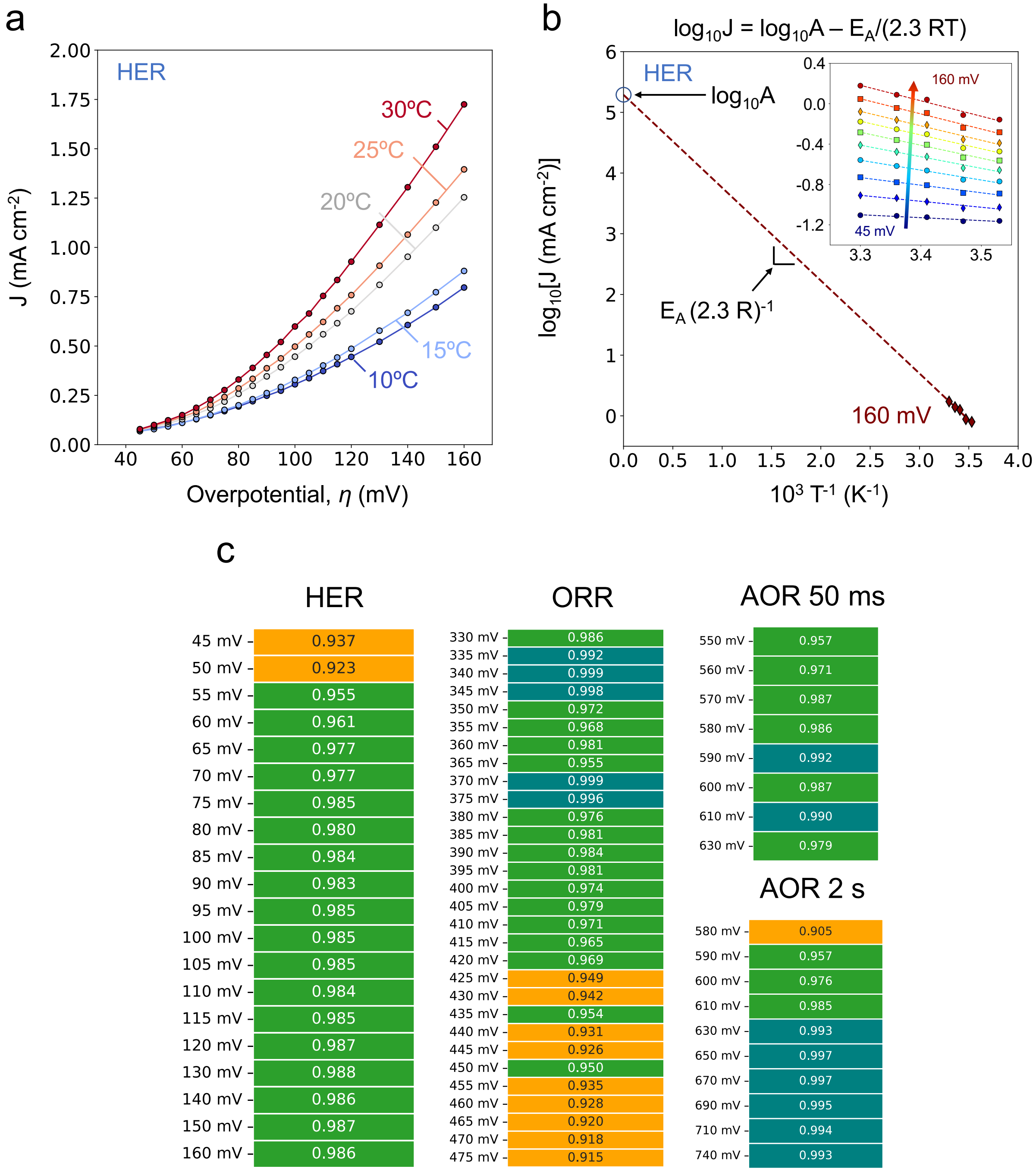

Supplementary Figure 1 | Temperature dependent Arrhenius analysis of the current density. **a,** HER chronoamperometric measurements at different temperatures. **b,** Arrhenius analysis of HER data to obtain A and E_A from the fit of the $\log_{10}$ of the measured current density against the inverse of the temperature in K^{-1}. E_A is obtained from the slope of the fit while A is obtained from the origin intercept extrapolating the regression to $1/T \rightarrow 0$. Note that R is the ideal gas constant defined as 8.314 J K^{-1} mol^{-1} **c**, Heatmap for R^2 values from linear Arrhenius fits for Figure 1**.** The R^2 value for all HER, ORR and AOR is generally well above 0.95, ensuring high accuracy of the reported E_A and A values. The colors in the table emphasize the R^2 of the extracted A and E_A.

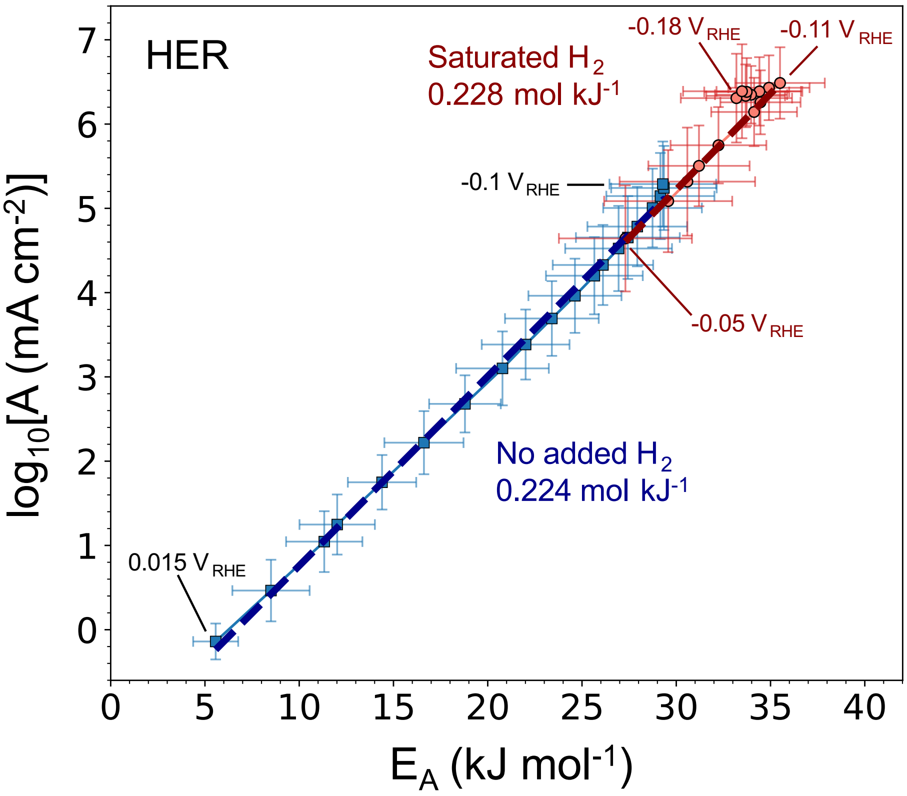

Supplementary Figure 2 | Impact of electrolyte H_2 concentration on HER kinetics. The slope is essentially independent of H_2 bubbling, whereas the absolute values of $\log_{10}A$ and E_A shift toward higher values. The kinetics start to deviate from the linear slope, due to the impact of the bias on the reaction enthalpy.

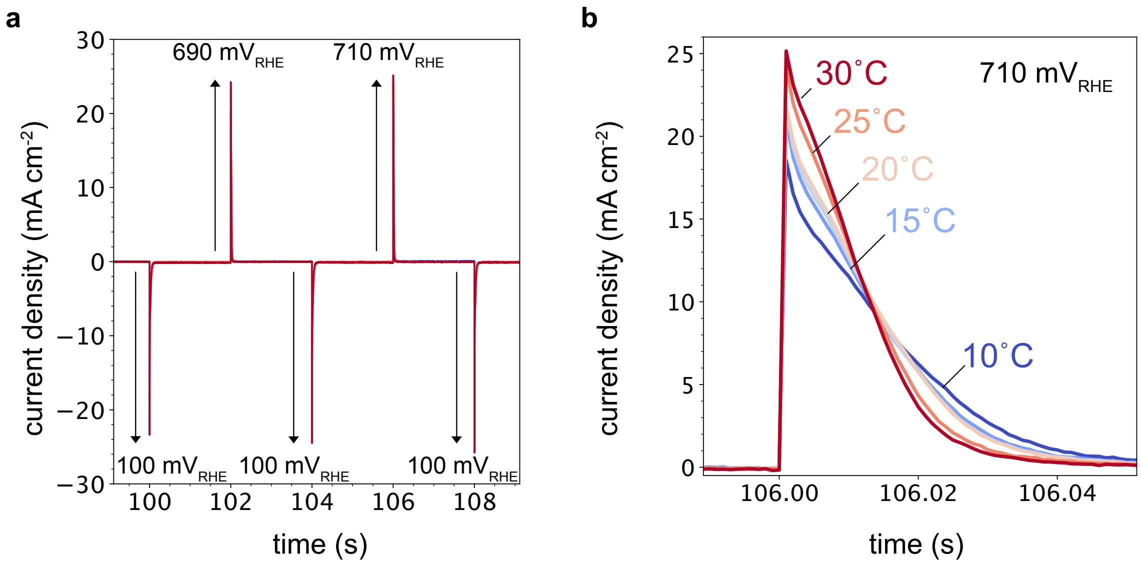

Supplementary Figure 3 | Temperature dependent potential jump studies on polycrystalline Pt foil in 0.1M KOH. a, Current density spikes during potential jumps as indicated. **b,** Temperature dependent current density transients for the first 40 ms after the jump to 710 mV_{RHE}. Such transients have been recorded throughout the whole potential range and Arrhenius analysis was applied.

	10 ms	15 ms	25 ms	30 ms
400 mV	0.954	0.913	0.471	0.490
425 mV	0.931	0.894	0.291	0.749
450 mV	0.924	0.881	0.705	0.889
475 mV	0.900	0.841	0.876	0.843
500 mV	0.923	0.645	0.883	0.773
525 mV	0.923	0.089	0.735	0.771
550 mV	0.904	0.047	0.719	0.776
575 mV	0.899	0.147	0.722	0.790
600 mV	0.910	0.283	0.762	0.842
610 mV	0.911	0.053	0.744	0.869
620 mV	0.911	0.174	0.797	0.871
630 mV	0.906	0.166	0.824	0.888
640 mV	0.926	0.345	0.855	0.908
650 mV	0.917	0.394	0.855	0.902
660 mV	0.931	0.326	0.867	0.905
670 mV	0.927	0.283	0.867	0.916
690 mV	0.905	0.469	0.891	0.906
710 mV	0.887	0.564	0.897	0.925
730 mV	0.874	0.593	0.914	0.916
750 mV	0.787	0.661	0.926	0.920
770 mV	0.663	0.697	0.938	0.905
800 mV	0.697	0.732	0.937	0.826

Supplementary Figure 4 | Heatmap for R^2 values from linear Arrhenius fits for Figure 2. The R^2 value for the Arrhenius fits extract from temperature dependent potential jump measurements in absence of NH_3 (Supplementary Figure 7). Generally, the R_2 values lower than for the steady state measurements in Supplementary Figure 1, but well above 0.7. To assess lower values, please see also Supplementary Note 2 and Supplementary Figure 15. The colors in the table emphasize the R^2 of the extracted A and E_A.

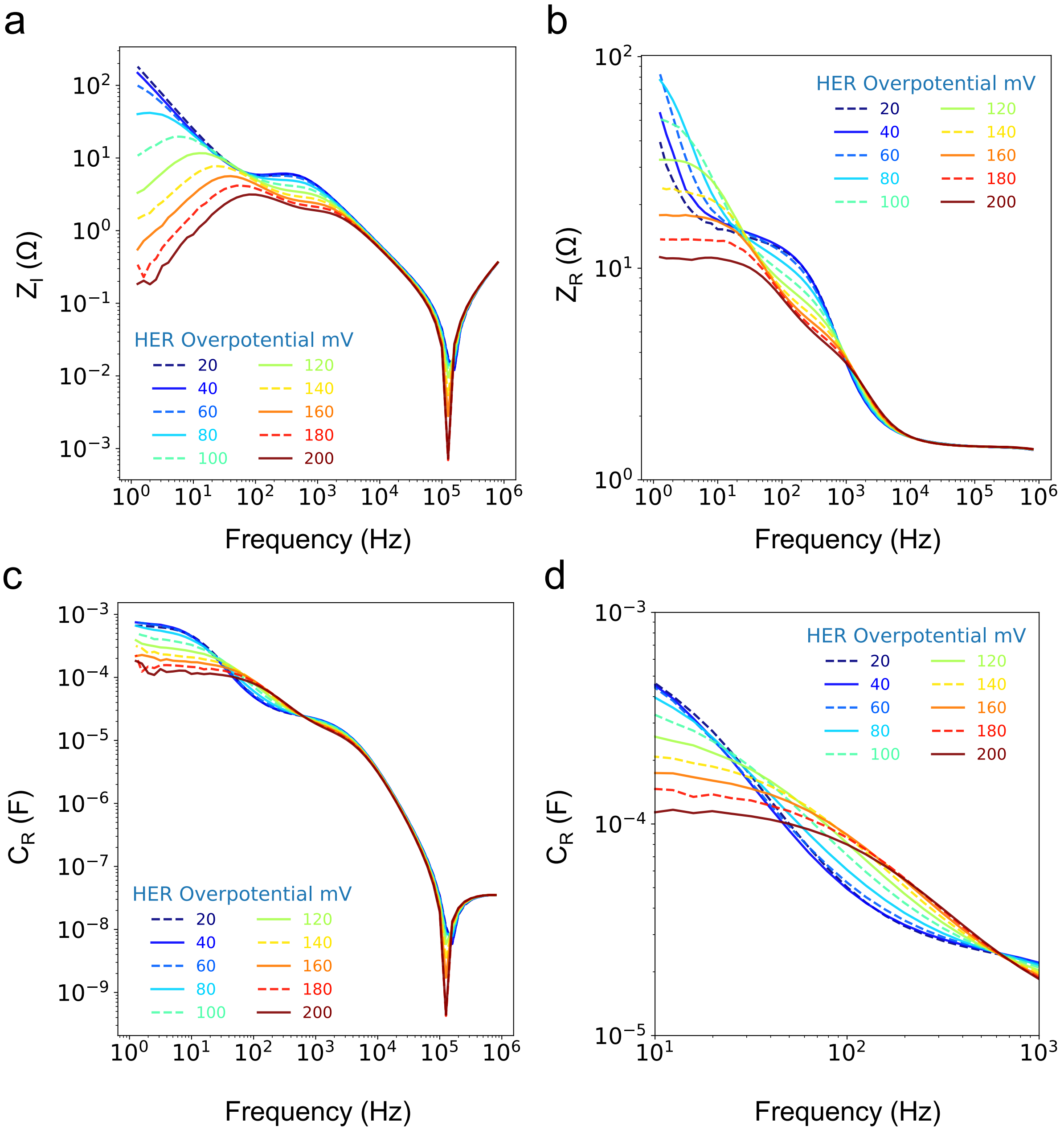

Supplementary Figure 5 | Impedance and Capacitance Bode plots for the HER. a-b, Potential dependent imaginary and real impedance Bode plots. Some noise is apparent at frequencies ~ < 10 Hz and potentials > 120 mV, which might be indicative of the start of (fluctuating) mass transport limitations caused by bubble formation. However, the noise does not impact the general trend with potential. Analysis of impedance > 50 kHz is excluded in the whole study, due to setup-related limitations (electrical connections). **c-d,** Potential dependent imaginary and real capacitance Bode plots, demonstrating a steady shift of the capacitance dispersion toward higher frequencies with increasing bias.

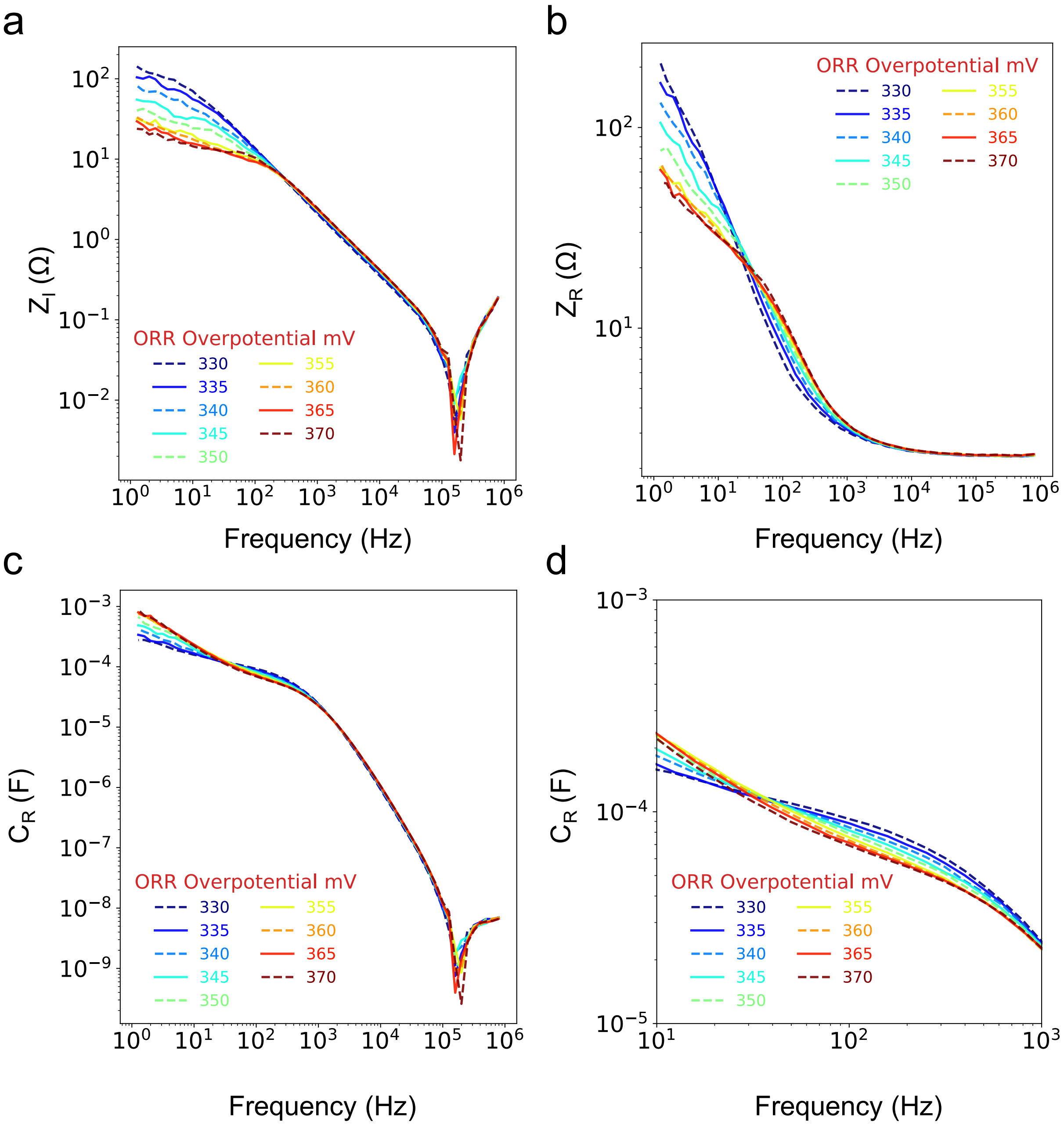

Supplementary Figure 6 | Impedance and Capacitance Bode plots for the ORR. a-b, Potential dependent imaginary and real impedance Bode plots. Some noise is apparent at frequencies ~ < 100 Hz and potentials > 330 mV, which might be indicative of the start of (fluctuating) mass transport limitations. However, the noise does not impact the general trend with potential. Analysis of impedance > 50 kHz is excluded in the whole study, due to setup related limitations. **c-d**, Potential dependent imaginary and real capacitance Bode plots, demonstrating a steady shift of the capacitance dispersion toward higher frequencies with increasing bias.

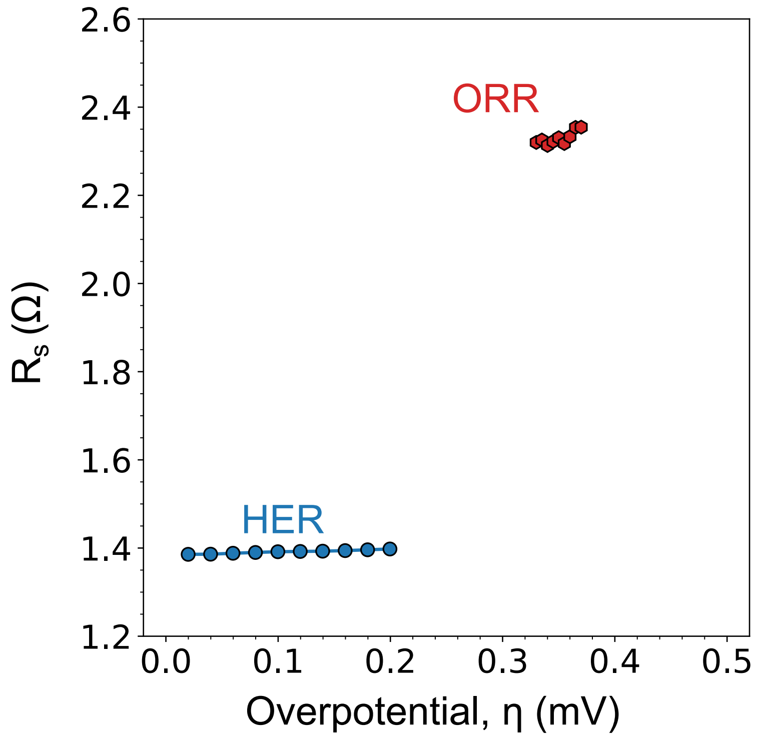

Supplementary Figure 7 | Series resistance for the HER and ORR. Potential dependent series resistance extracted from the high frequency limit of the real impedance in Bode plots. Note that there are no substantial potential dependent changes, highlighting the absence of any ionic or electronic transport limitations in the studied potential range.

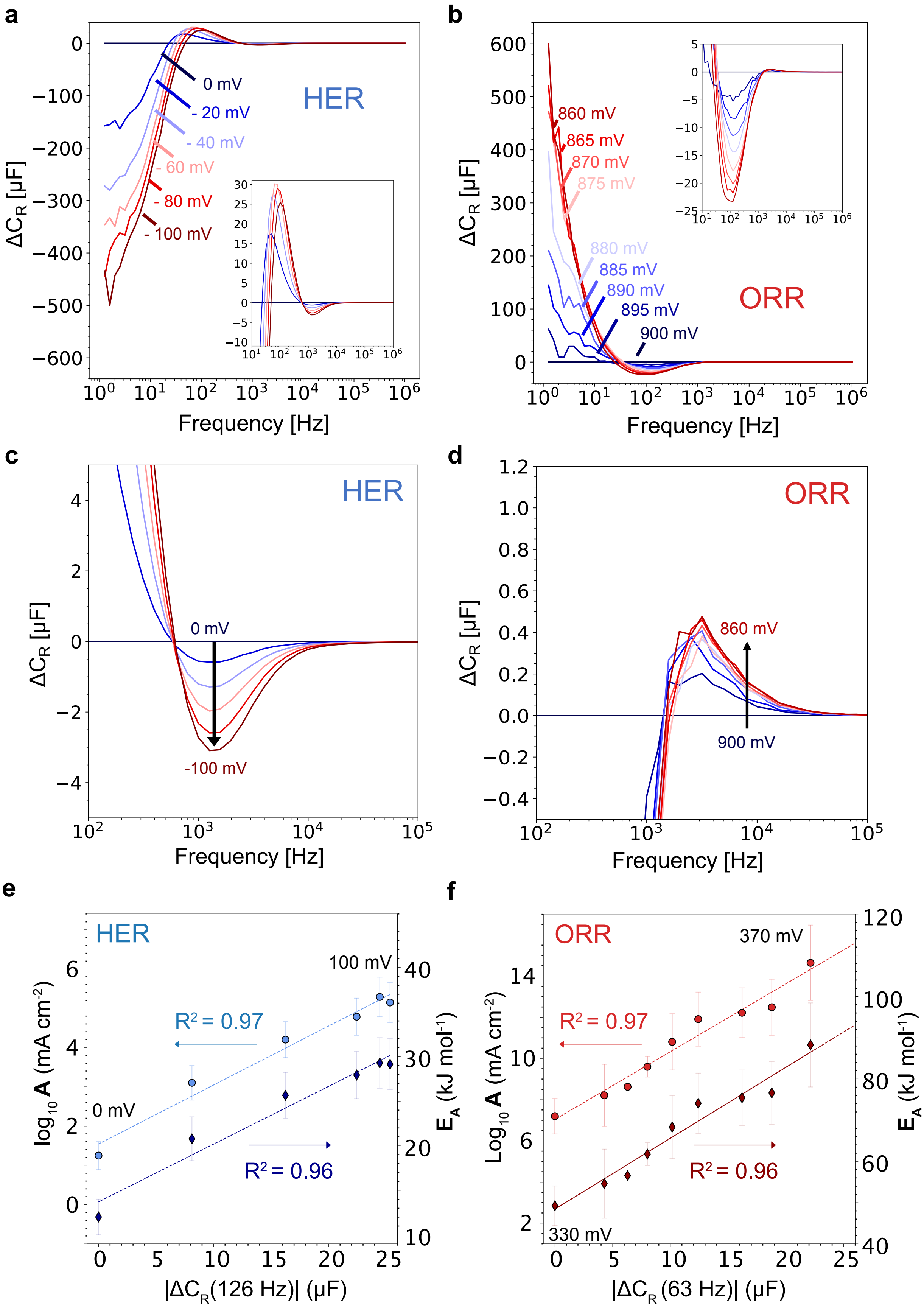
a
HER
0 mV
- 20 mV
- 40 mV
- 60 mV
- 80 mV
- 100 mV
ΔC_R [μF]
Frequency [Hz]
b
ORR
860 mV
865 mV
870 mV
875 mV
880 mV
885 mV
890 mV
895 mV
900 mV
ΔC_R [μF]
Frequency [Hz]
c
HER
0 mV
-100 mV
ΔC_R [μF]
Frequency [Hz]
d
ORR
860 mV
900 mV
ΔC_R [μF]
Frequency [Hz]
e
HER
100 mV
0 mV
R² = 0.97
R² = 0.96
log10 A (mA cm-2)
E_A (kJ mol-1)
|ΔC_R(126 Hz)| (μF)
f
ORR
370 mV
330 mV
R² = 0.97
R² = 0.96
Log10 A (mA cm-2)
E_A (kJ mol-1)
|ΔC_R (63 Hz)| (μF)

Supplementary Figure 8 | Bias dependent changes in the real capacitance for the HER and ORR. **a-b**, Potential (vs. RHE) dependent changes in the real capacitance for the HER and ORR. Strikingly, for both reactions, the changes in the capacitance with bias reach essentially the same limiting values (see also insets). These changes correlate strongly with the bias dependent pre-exponential factor. **c-d**, Magnified region of the potential dependent changes of the capacitance between 0.5-100kHz. These frequencies might be related to bias dependent ordering of interfacial (shuttle) water molecules or electrosorption, e.g. during the onset of PtO_x formation (in the case of the ORR). **e-f,** For the HER and ORR, changes in the pre-exponential factor and activation energy correlate directly with changes of the bias dependent real capacitance $|\Delta C_R(\nu, \eta)|$ (here exemplified at 126 Hz) that we associate with pseudo-capacitive electrosorption processes and double layer charging. **d**, For the ORR, changes in $A(\eta)$ and $E_A(\eta)$ correlate directly with $|\Delta C_R(\nu, \eta)|$ (shown at 63 Hz).

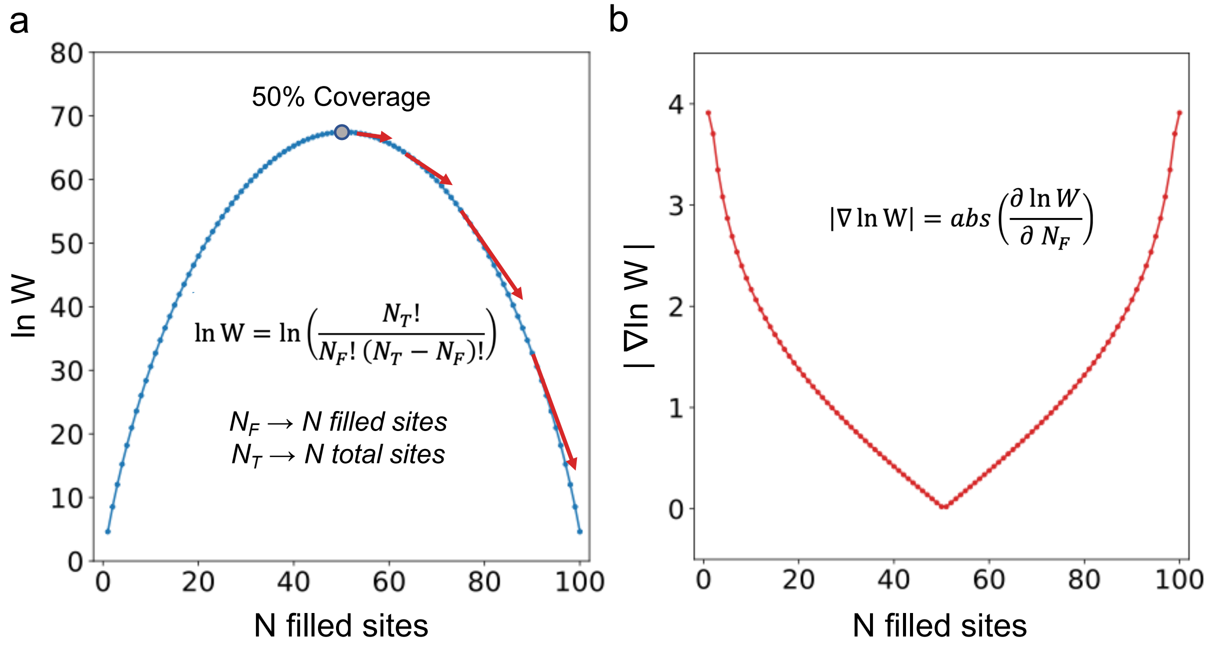

Supplementary Figure 9 | Configurational Entropy Changes. a, The maximum of the configurational entropy is obtained at a 50% coverage of all available (total) sites (N_T). Thus, with increasing or decreasing coverage (free sites N_F) the configurational entropy is reduced. The larger the reduction of configurational entropy upon occupation of a free site, the larger the pre-exponential factor. **b,** This incremental decrease in configurational entropy increases with increasing and decreasing initial coverage. Thus, for strongly covered or almost empty surfaces, the changes in the configurational entropy and, thus, the pre-exponential factor, can be substantial. However, as discussed throughout the manuscript, configurational entropy changes are not sufficient to explain the total change in A with bias. Further, they cannot explain the link with capacitance.

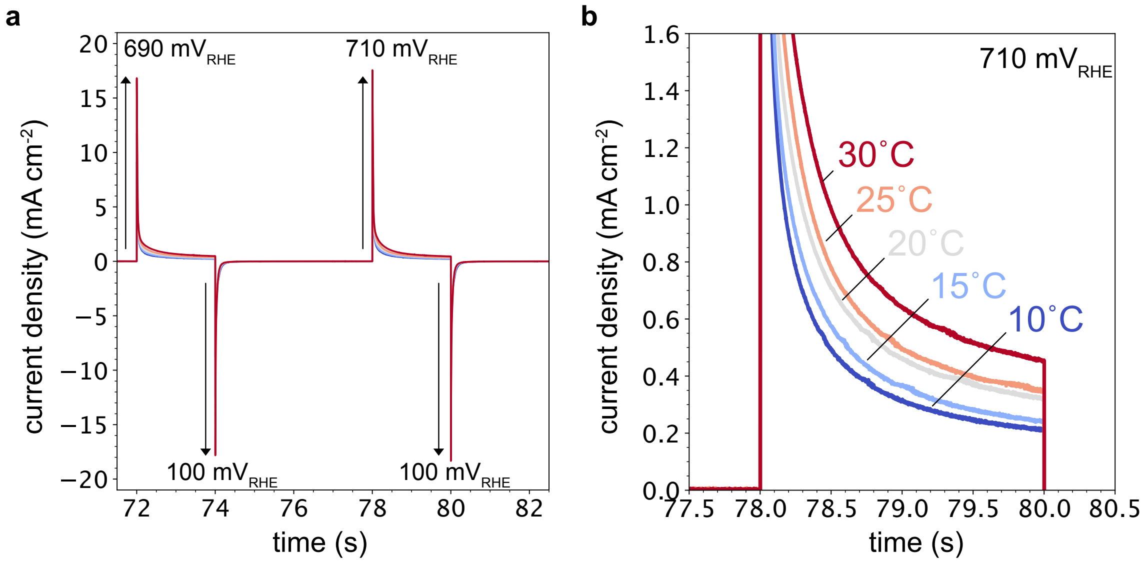

Supplementary Figure 10 | Temperature dependent potential jump studies on polycrystalline Pt foil in 0.1M KOH and with 0.1M NH_3. a, Current density spikes during potential jumps as indicated. **b,** Temperature dependent current density transients for 2s after the jump from 100 mV_{RHE} to 710 mV_{RHE}. These transients have been recorded throughout the whole potential range and Arrhenius analysis was applied.

	50 ms	100 ms	200 ms	500 ms	2000 ms
600 mV	0.644	0.948	0.872	0.865	0.887
610 mV	0.218	0.966	0.901	0.938	0.957
620 mV	0.055	0.976	0.907	0.953	0.971
630 mV	0.670	0.978	0.949	0.977	0.987
640 mV	0.905	0.983	0.966	0.986	0.986
650 mV	0.957	0.986	0.977	0.993	0.992
660 mV	0.976	0.989	0.989	0.996	0.987
670 mV	0.985	0.992	0.992	0.997	0.990
690 mV	0.993	0.994	0.996	0.993	0.979
710 mV	0.997	0.996	0.997	0.986	0.971
730 mV	0.997	0.997	0.995	0.980	0.961
750 mV	0.995	0.997	0.990	0.968	0.963
770 mV	0.994	0.998	0.976	0.945	0.937
800 mV	0.993	0.997	0.932	0.924	0.898

Supplementary Figure 11 | Heatmap for R^2 values from linear Arrhenius fits for Figure 3. The R^2 value for all AOR data is generally well above 0.9, ensuring high accuracy of the reported E_A and A values. Only at 50 ms and potentials < 650 mV the fits become less reliable. The colors in the table emphasize the R^2 of the extracted A and E_A.

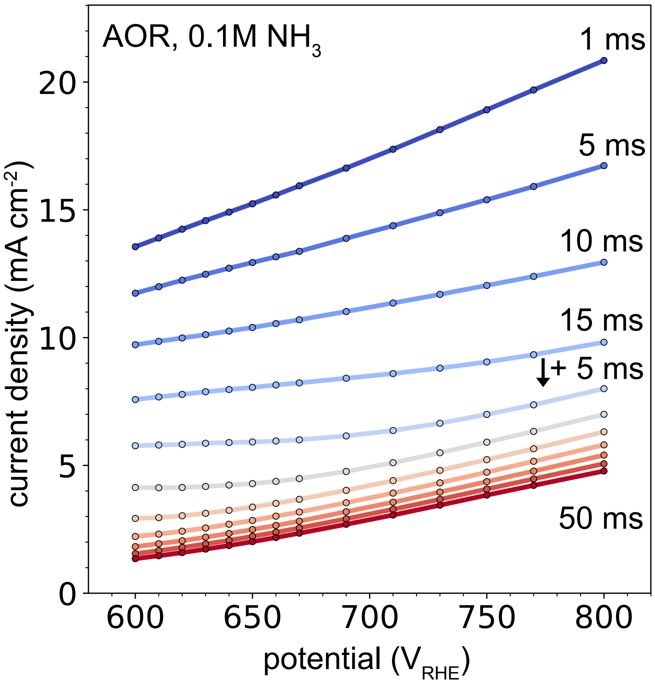

Supplementary Figure 12 | AOR discharge shortly after potential pulse (≤ 50 ms) in 0.1M KOH on polycrystalline Pt. Except for very short times, Faradaic current arises clearly even after 10-15 ms. At times ≥ 50 ms, the response is largely dominated by Faradaic AOR current.

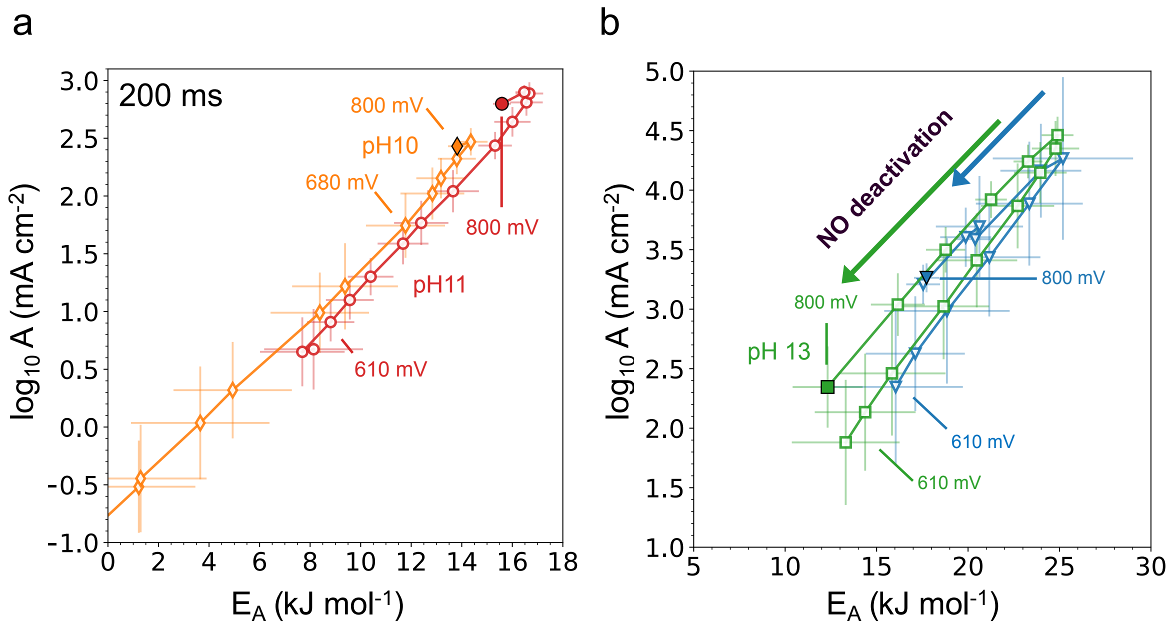

Supplementary Figure 13 | Kinetic E_A-A map for AOR at 200 ms. a-b, Changes in E_A and A as a function of pH for 200 ms after the potential jump. NO deactivation is almost absent at lower pH, however, log A reaches maximum values of ~ 2.5. In contrast, at higher pH, the maximum of the pre-exponential factor increases for lower bias by almost two orders of magnitude, but *NO deactivation decreases the pre-exponential factor again to similar values as for the lower pH values. This is indicative of capacitive discharge or electronic passivation and deactivation of the Pt surface. All E_A and A are extracted from Arrhenius fits with R^2 values given in Supplementary Figure 14.

40 ms

	pH10	pH11	pH12	pH13
610 mV	0.874	0.846	0.893	0.809
620 mV	0.899	0.884	0.928	0.701
630 mV	0.919	0.908	0.831	0.431
640 mV	0.933	0.925	0.926	0.019
650 mV	0.943	0.943	0.481	0.247
660 mV	0.945	0.950	0.009	0.652
670 mV	0.944	0.950	0.486	0.850
690 mV	0.900	0.913	0.738	0.963
710 mV	0.076	0.649	0.985	0.997
730 mV	0.924	0.054	0.980	0.996
750 mV	0.977	0.798	0.989	0.995
770 mV	0.995	0.933	0.986	0.993
800 mV	0.999	0.976	0.989	0.990

200 ms

	pH10	pH11	pH12	pH13
610 mV	0.060	0.876	0.864	0.901
620 mV	0.090	0.967	0.944	0.907
630 mV	0.076	0.971	0.930	0.949
640 mV	0.372	0.978	0.910	0.966
650 mV	0.597	0.978	0.950	0.977
660 mV	0.860	0.978	0.955	0.989
670 mV	0.870	0.983	0.935	0.992
690 mV	0.950	0.994	0.975	0.996
710 mV	0.972	0.994	0.993	0.997
730 mV	0.984	0.995	0.961	0.995
750 mV	0.991	0.997	0.985	0.990
770 mV	0.994	0.999	0.992	0.976
800 mV	0.998	0.998	0.996	0.932

Supplementary Figure 14 | R^2 values of Arrhenius fits for Figure 4 in the main manuscript. The R^2 value for all AOR data is generally well above 0.8. To assess lower values, please see also Supplementary Note 2 and Supplementary Figure 15. However, especially at 40 ms, multiple fits are less reliable. The colors in the table emphasize the R^2 of the extracted A and E_A.

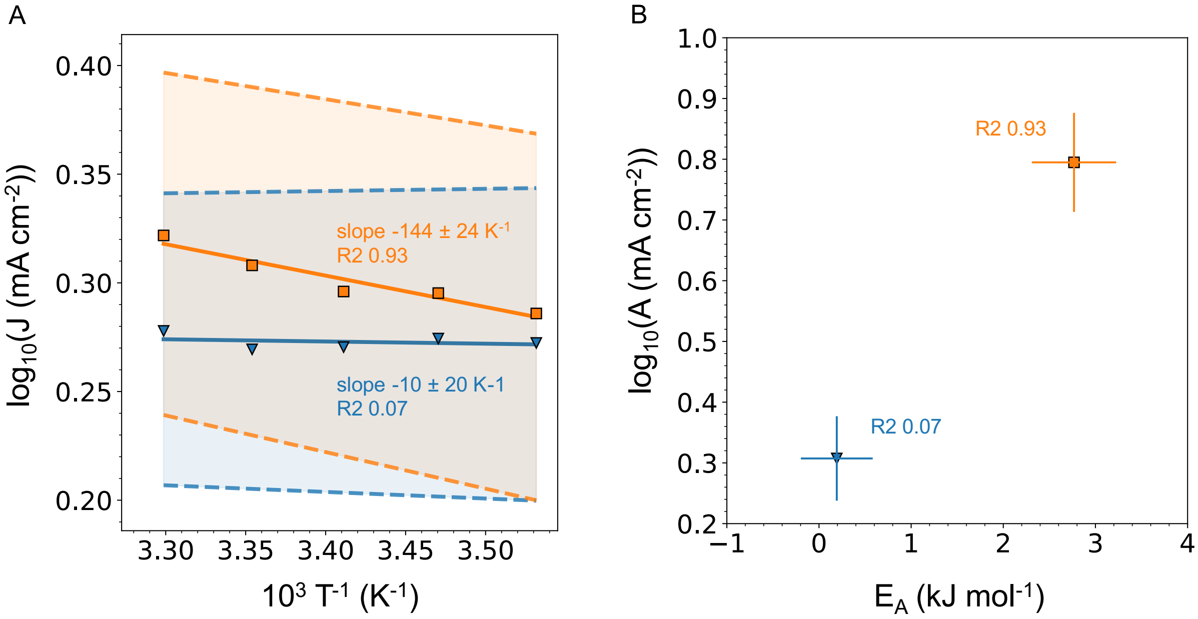

Supplementary Figure 15 | Comparison of goodness of fits for two linear regressions showing the difference between R^2 and S. **a,** The orange line has a high R^2 coefficient, while the absolute standard error of the slope is comparable to the blue line which displays a low R^2 coefficient since the blue data points have a small variation of the current density with the temperature (small magnitude of the activation energy). **b,** Both linear fits result in two points with similar error bars despite having largely different R^2 coefficients.

Supplementary References

1. Bockris, J. O., Gileadi, E. & Müller, K. Dielectric Relaxation in the Electric Double Layer. *The Journal of Chemical Physics* **44**, 1445–1456 (1966).
2. Santos, E., Aradi, B., van der Heide, T. & Schmickler, W. Free energy curves for the Volmer reaction obtained from molecular dynamics simulation based on quantum chemistry. *Journal of Electroanalytical Chemistry* **954**, 118044 (2024).
3. Sarabia, F. J., Sebastián-Pascual, P., Koper, M. T. M., Climent, V. & Feliu, J. M. Effect of the Interfacial Water Structure on the Hydrogen Evolution Reaction on Pt(111) Modified with Different Nickel Hydroxide Coverages in Alkaline Media. *ACS Applied Materials & Interfaces* **11**, 613–623 (2019).
4. Climent, V., Coles, B. A. & Compton, R. G. Coulostatic potential transients induced by laser heating of a Pt(111) single-crystal electrode in aqueous acid solutions. Rate of hydrogen adsorption and potential of maximum entropy. *Journal of Physical Chemistry B* **106**, 5988–5996 (2002).
5. Bazant, M. Z., Thornton, K. & Ajdari, A. Diffuse-charge dynamics in electrochemical systems. *Phys. Rev. E* **70**, 021506 (2004).
6. Santos, E. & Schmickler, W. On the timescale of electrochemical processes. *Electrochimica Acta* 144659 (2024) doi:10.1016/j.electacta.2024.144659.
7. Ojha, K., Arulmozhi, N., Aranzales, D. & Koper, M. T. M. Double Layer at the Pt(111)–Aqueous Electrolyte Interface: Potential of Zero Charge and Anomalous Gouy–Chapman Screening. *Angewandte Chemie International Edition* **59**, 711–715 (2020).
8. Ojha, K., Doblhoff-Dier, K. & Koper, M. T. M. Double-layer structure of the Pt(111)–aqueous electrolyte interface. *Proceedings of the National Academy of Sciences* **119**, 1–9 (2022).
9. Schmickler, W. & Santos, E. Desorption of Hydrogen from Graphene Induced by Charge Injection. *ChemElectroChem* **9**, 1–8 (2022).
10. Rodellar, C. G., Gisbert-Gonzalez, J. M., Sarabia, F., Roldan Cuenya, B. & Oener, S. Z. Ion solvation kinetics in bipolar membranes and at electrolyte–metal interfaces. *Nat Energy* (2024) doi:10.1038/s41560-024-01484-z.
11. Wang, S. *et al.* Electrochemical impedance spectroscopy. *Nature Reviews Methods Primers* **1**, 41 (2021).
12. Taberna, P. L., Simon, P. & Fauvarque, J. F. Electrochemical Characteristics and Impedance Spectroscopy Studies of Carbon-Carbon Supercapacitors. *Journal of The Electrochemical Society* **150**, A292 (2003).
13. Sibert, E., Faure, R. & Durand, R. High frequency impedance measurements on Pt(111) in sulphuric and perchloric acids. *Journal of Electroanalytical Chemistry* **515**, 71–81 (2001).
14. Scohy, M. *et al.* Investigating the oxygen evolution reaction on Ir(111) electrode in acidic medium using conventional and dynamic electrochemical impedance spectroscopy. *Electrochimica Acta* **320**, 134536 (2019).
15. Schouten, K. J. P., Van Der Niet, M. J. T. C. & Koper, M. T. M. Impedance spectroscopy of H and OH adsorption on stepped single-crystal platinum electrodes in alkaline and acidic media. *Phys. Chem. Chem. Phys.* **12**, 15217 (2010).
16. Lewis, N. B., Bisbey, R. P., Westendorff, K. S., Soudackov, A. V. & Surendranath, Y. A molecular-level mechanistic framework for interfacial proton-coupled electron transfer kinetics. *Nat. Chem.* **16**, 343–352 (2024).
17. Kuo, D.-Y., Lu, X., Hu, B., Abruña, H. D. & Suntivich, J. Rate and Mechanism of Electrochemical Formation of Surface-Bound Hydrogen on Pt(111) Single Crystals. *J. Phys. Chem. Lett.* **13**, 6383–6390 (2022).
