# Supplementary material for: Exploring dynamic solvation kinetics at electrocatalyst surfaces
Source: Nat Commun. 2024 Sep 18;15:8204. doi: 10.1038/s41467-024-52499-9 (PMC11411097; doi:10.1038/s41467-024-52499-9)
Supplement: Supplementary file 3 — Description of Additional Supplementary Files [file 41467_2024_52499_MOESM3_ESM.pdf]

Description of Additional Supplementary Files

File Name: Supplementary Movie 1
Description: Dynamic Solvation Kinetics during Electrosorption at pH 13.

File Name: Supplementary Movie 2
Description: Dynamic Solvation Kinetics during Ammonia Oxidation at pH 13.

File Name: Supplementary Movie 3
Description: Dynamic Solvation Kinetics during Ammonia Oxidation at pH 12.

File Name: Supplementary Movie 4
Description: Dynamic Solvation Kinetics during Ammonia Oxidation at pH 11.

File Name: Supplementary Movie 5
Description: Dynamic Solvation Kinetics during Ammonia Oxidation at pH 10.
